# Abnormal pro-gly-pro pathway and airway neutrophilia in pediatric cystic fibrosis

**DOI:** 10.1016/j.jcf.2019.05.017

**Published:** 2020-01

**Authors:** Andrew R. Turnbull, Chloe J. Pyle, Dhiren F. Patel, Patricia L. Jackson, Tom N. Hilliard, Nicolas Regamey, Hui-Leng Tan, Sarah Brown, Rebecca Thursfield, Christopher Short, Megan Mc Fie, Eric W.F.W. Alton, Amit Gaggar, J. Edwin Blalock, Clare M. Lloyd, Andrew Bush, Jane C. Davies, Robert J. Snelgrove

**Affiliations:** a Cystic Fibrosis and Chronic Lung Infection, National Heart & Lung Institute, Imperial College London, United Kingdom; b Paediatric Respiratory Medicine, Royal Brompton & Harefield NHS Foundation Trust, London, United Kingdom; c Inflammation Repair and Development, National Heart and Lung Institute, Imperial College London, London SW7 2AZ, United Kingdom; d Division of Pulmonary, Allergy and Critical Care Medicine, Program in Protease and Matrix Biology, Gregory Fleming James Cystic Fibrosis Centre and Lung Health Center, University of Alabama at Birmingham, Birmingham, AL 35294, United States of America; e Birmingham V.A. Medical Centre, Birmingham, AL 35294, United States of America; f Paediatric Respiratory Medicine, Children's Hospital, Lucerne, Switzerland; g Alder Hey Children's NHS Foundation Trust, Liverpool L14 5AB, United Kingdom

**Keywords:** Neutrophil, Protease, Matrikine, Cystic fibrosis

## Abstract

**Background:**

Proline–glycine–proline (PGP) is a bioactive fragment of collagen generated by the action of matrix metalloproteinase-9 (MMP-9) and prolylendopeptidase (PE), and capable of eliciting neutrophil chemotaxis and epithelial remodelling. PGP is normally then degraded by leukotriene A_4_ hydrolase (LTA_4_H) to limit inflammation and remodelling. This study hypothesized that early and persistent airway neutrophilia in Cystic Fibrosis (CF) may relate to abnormalities in the PGP pathway and sought to understand underlying mechanisms.

**Methods:**

Broncho-alveolar lavage (BAL) fluid was obtained from 38 CF (9 newborns and 29 older children) and 24 non-CF children. BAL cell differentials and levels of PGP, MMP-9, PE and LTA_4_H were assessed.

**Results:**

Whilst PGP was present in all but one of the older CF children tested, it was absent in non-CF controls and the vast majority of CF newborns. BAL levels of MMP-9 and PE were elevated in older children with CF relative to CF newborns and non-CF controls, correlating with airway neutrophilia and supportive of PGP generation. Furthermore, despite extracellular LTA_4_H commonly being greatly elevated concomitantly with inflammation to promote PGP degradation, this was not the case in CF children, potentially owing to degradation by neutrophil elastase.

**Conclusions:**

A striking imbalance between PGP-generating and -degrading enzymes enables PGP accumulation in CF children from early life and potentially supports airway neutrophilia.

## Highlights

• PGP is a collagen-derived pro-neutrophilic mediator.
• PGP accumulates in the airways of children with cystic fibrosis.
• Imbalance of PGP generation (MMP/PE) vs degradation (LTA_4_H) enables PGP build-up.
• LTA_4_H is destroyed by neutrophil elastase to prevent PGP degradation.
• Infections are key to spiking PGP generation in setting it cannot be degraded.

## Graphical abstract

**Figure:**
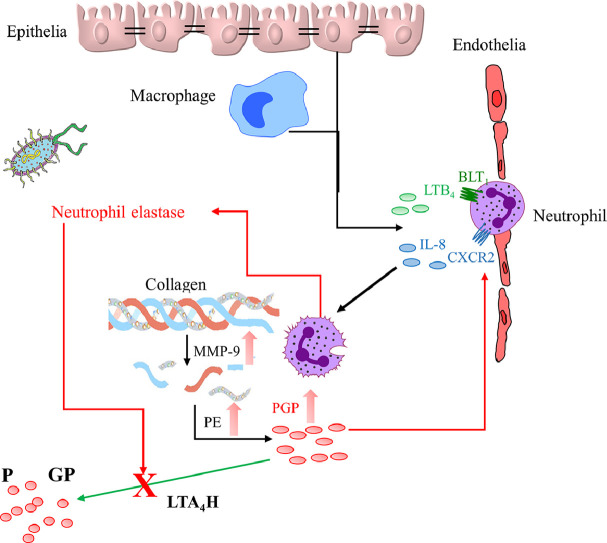

## 1 Introduction

Neutrophils are critical components of the body's immune response to infection, but owing to the indiscriminate nature of their products an over-exuberant or persistent neutrophilic inflammation is implicated in the pathologies of chronic airways diseases such as chronic obstructive pulmonary disease (COPD) and cystic fibrosis (CF). A protease-antiprotease imbalance is a hallmark of these diseases, whereby several matrix metalloproteinases (MMPs) and neutrophil elastase (NE) correlate with pathological changes. Proteases degrade components of the extracellular matrix (ECM), disrupting tissue architecture and releasing ECM-derived chemoattractant signals, termed ‘matrikines’, that perpetuate inflammation.

The tripeptide Proline-Glycine-Proline (PGP) is one such matrikine [1,2], being a neutrophil chemoattractant derived from ECM collagen via the sequential enzymatic activity of specific matrix metalloproteinases (MMPs) and prolylendopeptidase (PE) [3]. PGP can subsequently be chemically acetylated on its N-terminus (Ac-PGP), which functions to enhance its chemotactic potential [4,5]. Since neutrophils are a source of the proteases that generate PGP it is anticipated that this pathway can drive a self-sustaining circle of neutrophilic inflammation [6]. Accordingly, PGP/AcPGP is found in patients with chronic airways diseases, correlating with disease severity and supporting neutrophilic persistence [1,3,7,8]. More recently, we have demonstrated PGP/AcPGP is also capable of directly promoting proliferation and radial spreading of bronchial epithelial cells, independent of its action on neutrophils [9]. PGP/AcPGP persistence was subsequently demonstrated to drive pathological epithelial remodelling by augmenting epithelial hypertrophy and mucus hypersecretion [9], which are again hallmark feature of specific chronic lung diseases.

To limit PGP persistence, we identified an anti-inflammatory pathway whereby PGP is degraded during episodes of acute neutrophilic inflammation by the extracellular activity of the enzyme leukotriene A_4_ hydrolase (LTA_4_H) [4]. We demonstrated that this degradation was critical to limit inflammation [10] and to prevent pathological epithelial remodelling [9]. LTA_4_H classically functions intracellularly to catalyse the generation of pro-inflammatory lipid mediator leukotriene B_4_ (LTB_4_) [11,12]. LTB_4_ can drive the recruitment and activation of an array of cells including neutrophils and is implicated in protection against micro-organisms but also in the pathology of multiple diseases [13]. Thus LTA_4_H represents a highly unusual enzyme with opposing pro- and anti-inflammatory activities that dictate the amplitude and persistence of neutrophilic inflammation [14].

CF is a lethal genetic disorder caused by defective function of the Cystic Fibrosis Transmembrane Conductance Regulator, which predispose patients to infection, chronic airway inflammation and airway remodelling. The airways of CF patients are thought to lack inflammation at birth, but become inflamed and infected with characteristic pathogens early in life [15]. Neutrophils are a prominent feature of CF airway inflammation, with numbers and activity exhibiting an inverse relationship with lung function [[16], [17], [18]]. We hypothesized that PGP could support neutrophilic inflammation in early life CF as a consequence of an aberrant LTA_4_H-PGP pathway. We subsequently demonstrate that BAL fluid in children with established CF contains PGP, correlating with neutrophilic inflammation, as a consequence of a striking imbalance between PGP-generating and –degrading enzymes, with the latter targeted for proteolytic degradation in the CF lung.

## 2 Methods

### 2.1 Subjects

Samples were obtained from patients undergoing clinically-indicated fibreoptic bronchoscopy, as previously described [19], in the Pediatric department of the Royal Brompton Hospital. BAL fluid was processed as described previously [20] and stored for research once all clinical tests had been undertaken. CF had been diagnosed on conventional criteria [21]. *CFTR* genotyping had been undertaken as part of clinical diagnosis by the local screening genetics centres. Those in the ‘routine screened infant surveillance programme’ (RSISP)-CF group had all been diagnosed as part of the UK Newborn Screening programme and were undergoing the Centre's routine surveillance investigations scheduled for around 4 months of life; the ‘clinical concern’ (CC)-CF group comprised of patients older than 4 months who were having a bronchoscopy because of clinical deterioration; this was a mix of medium-term concerns and acute (symptomatic) exacerbation, but as there was substantial overlap between these groups, further subdivision has not been undertaken. Indications for bronchoscopy within the non-CF controls were as defined in Table 1. Microbiological culture status was prospectively defined as positive if any airway sample grew bacteria or fungi at the time of the bronchoscopy or in the previous two weeks. Data were not available for viral detection. Spirometric data were collected, where available, from the CF annual assessments for the 5 years following bronchoscopy; in our centre, these measurements are only made on children from around 5 years of age. The research project was approved by the Royal Brompton & Harefield Research Ethics Committee and the institution's Respiratory Biomedical Research Unit under project 10/H0504/9. All parents gave informed consent; age-appropriate assent was also sought.

**Table 1:** Demographics of CF and control children undergoing bronchoscopy.

	Non-CF control (n = 24)	RSISP-CF (n = 9)	CC-CF (n = 29)
Male: Female (%)	33: 67	50: 50	45: 55
Age, years	6.5 (10.6)	0.3 (0.2)	11.9 (5.8)
CFTR genotype (n)	ND	ΔF_508_ / ΔF_508_ = 5 ΔF_508_ / Other = 3 Other / Other = 1	ΔF_508_ / ΔF_508_ = 16 ΔF_508_ / Other = 10 Other / Other = 1 Unknown = 2
Indication for bronchoscopy (n)	Stridor (n = 4); Recurrent croup (n = 3); Broncho/Laryngomalacia (n = 6); Haemoptysis (n = 4); Persistent dry cough (n = 2); Barking cough (n = 1); Vascular ring (n = 1); Recurrent chest infections (n = 1); Breathing difficulties (n = 2)	Routine surveillance as part of newborn screening programme	Clinical deterioration or loss of lung function
Culture positive (%)	33%	11%	79%
Pathogens in BAL fluid	Bacteria *P. aeruginosa* (n = 1) *S. aureus* (n = 5) *Other* (n = 3)	Bacteria *P.aeruginosa* (n = 1)	Bacteria *P. aeruginosa* (n = 12) *S. aureus* (n = 5) *Other* (n = 4) Fungi *A. fumigatus* (n = 12) *C. albicans* (n = 4)

Data presented as median value (IQR) unless otherwise stated.

BAL, bronchoalveolar lavage; RSISP, routine screened infant surveillance program; CC, bronchoscopy for clinical concern; ND, not determined.

### 2.2 Protease quantification and activity

The concentration of MMP-9 (R&D Systems, Minneapolis, MN), PE (MyBiosource, San Diego, CA, USA) and LTA_4_H (USCN Life Science, Hubei, PRC) in BAL fluid was measured using an ELISA, according to the manufacturer's directions. BAL fluid MMP-9 activity was determined by Fluorokine E kit (R&D Systems, Minneapolis, MN), and NE activity by a Fluorometric assay kit (Abcam, Cambridge, UK), according to the manufacturer's directions.

### 2.3 Interleukin-8 (IL-8) quantification

The concentration of IL-8 in BAL fluid was measured using an ELISA, according to the manufacturer's directions (R&D Systems, Minneapolis, MN).

### 2.4 LTB_4_ quantification

The concentration of LTB_4_ in BAL fluid was assayed using an ELISA, according to manufacturer's directions (R&D systems, Minneapolis, MN).

### 2.5 PGP interrogation

BAL fluid concentrations of PGP/AcPGP were determined by liquid chromatography-electrospray ionization-tandem mass spectrometry (ESI-LC/MS/MS), as previously described [10]. To ascertain the PGP-degrading activity of BAL fluid, it was incubated with 0.4 mM PGP at 37 °C in 5% CO_2_ for varying periods of time. Degradation was subsequently calculated my enumeration of loss of peptide by ESI-LC/MS/MS and release of free proline and its ensuing reaction with Ninhydrin, as previously described [10] (additional details provided in an online data supplement).

### 2.6 LTA_4_H degradation by NE

Recombinant human LTA_4_H (final concentration 20-100 μg/ml; Cayman Chemical, Ann Arbor, USA) and NE (final concentration 20-100 μg/ml; Abcam, Cambridge, UK) were incubated alone or in combination in a final volume of 100 μl PBS at 37 °C in 5% CO_2_ for 2 h and LTA_4_H protein levels determined via Coomassie staining of gels and Western blot. Ensuing LTA_4_H PGP-degrading activity was assessed by ESI-LC/MS/MS and generation of free proline as described above (additional details provided in an online data supplement).

### 2.7 Statistical analysis

There were no previous data to inform a power calculation, so group size is opportunistic based on availability of samples. For nonparametric data, shown as median (IQR), between-group comparisons were performed with a Kruskal-Wallis test followed by a Dunns post-test. Associations were tested by Spearman rank correlation.

## 3 Results

### 3.1 Patient demographics

BAL fluid was analysed from 62 children: 24 non-CF controls, 29 in the CC-CF group and 9 in the RSISP-CF group (Table 1). Indications for bronchoscopy within the non-CF controls were as defined in Table 1. Of the non-CF controls, 8 were culture positive (*P. aeruginosa* (n = 1); *M. catarrhails* (n = 1); coliforms (n = 1); *S. aureus* (n = 4); *H. influenzae* and *S. pneumoniae* (n = 1)). The majority of the CC-CF group (23/29, 79% patients) were culture positive. The RSISP-CF group were culture negative (n = 8, 89%) with the exception of one child who was culture positive for *P. aeruginosa.* (Table 1). Accordingly, this cohort of patients provided an excellent opportunity to retrospectively assess the effect of infection and CF status, alone and in combination, upon the PGP pathway. 18 (62%) CC-CF patients had spirometry available from the annual assessment within the year following bronchoscopy (mean (SD) % predicted FEV_1_ = 73.4 (17.2); mean (SD) % predicted FVC = 83.5 (16.1)). 21 (72%) CC-CF patients had at least 3 annual spirometry values during the 5 years following bronchoscopy, enabling annual change in FEV_1_ to be calculated.

### 3.2 Elevated neutrophilic inflammation in the BAL fluid of CC-CF children

BAL absolute cell counts and differentials were available respectively in 19 (79%) and 23 (96%) of the non-CF controls, 20 (69%) of CC-CF patients and 8 (89%) of the RSISP-CF patients (Table 2). As expected, BAL fluid from the CC-CF group contained more neutrophils than non-CF controls and RSISP-CF patients (Table 2). Whilst macrophages were prominent component of the infiltrate in the BAL fluid of all children, total numbers were not significantly different between groups. Numbers of lymphocytes and eosinophils were modest but a greater number of eosinophils were present in the CC-CF group versus non-CF controls. No statistically significant differences were observed between non-CF controls and RSISP-CF patients in total numbers of any cell population.

**Table 2:** Total and differential inflammatory cell counts in bronchoalveolar lavage (BAL) fluid.

	Non-CF control (n = 24)	RSISP-CF (n = 9)	CC-CF (n = 29)
Absolute cells x10^3^ (/ml)	270 (235)	386 (453.8)	976.5 (2516.3)**
Macrophages (%)	87 (20)	82.5 (16.1)	49.7 (60)***^
Macrophages x10^3^ (/ml)	158.4 (145.7)	428.6 (337.4)	191.1 (387.7)
Neutrophils (%)	1 (5.7)	11.2 (23.7)	44.7 (56.4)**^
Neutrophils x10^3^ (/ml)	3.3 (29.8)	57.7 (101.1)	528 (1755.1)***^
Eosinophils (%)	0.3 (0.3)	0.3 (0.7)	0.9 (4)*
Eosinophils x10^3^ (/ml)	0.2 (1)	1.9 (3.1)	17.9 (31.2)**
Lymphocytes (%)	3.3 (8.7)	6 (7.2)	1.7 (5.3)
Lymphocytes x10^3^ (/ml)	7.3 (17.5)	33 (43.4)	30.8 (49.6)

Data presented as median value (IQR) unless otherwise stated.

Statistical significance between groups was tested using a Kruskal-Wallis test followed by a Dunns post-test. * = between non-CF controls vs CC children (* ≤0.05; ** ≤0.01; *** ≤0.001); ^ = between RSISP vs CC children (^ ≤0.05).

### 3.3 PGP is elevated in the BAL fluid of CC-CF children

**Fig. 1:**
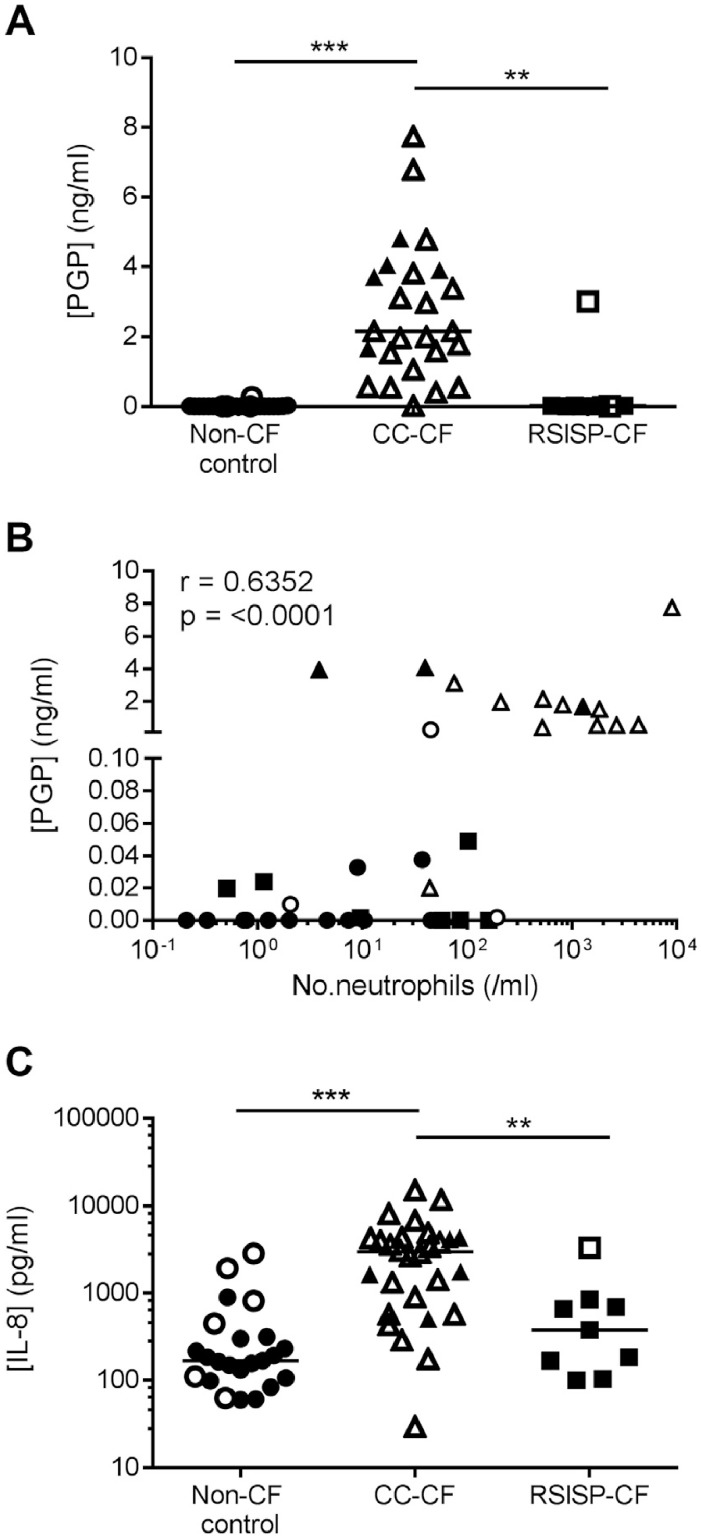
The neutrophil matrikine PGP is elevated in the BAL fluid of older children with CF. (A) Levels of PGP peptide in the BAL fluid of non-CF controls (n = 24), RSISP-CF children (n = 9) and CC-CF children (n = 25), as determined by LC-MS/MS. (B) Correlation between levels of BAL fluid PGP and airway neutrophils (n = 37: 16 non-CF, 7 RSISP-CF, 14 CC-CF). (C) Levels of IL-8 in the BAL fluid of non-CF controls (n = 23), RSISP-CF children (n = 9) and CC-CF children (n = 28), as determined by ELISA. Closed symbols represent patients that were culture negative and open symbols patients that were culture positive. The horizontal bar depicts the median of each group. Statistical significance between groups was tested using a Kruskal-Wallis test followed by a Dunns post-test and correlation analysis was performed using Spearman rank test. * = P < .05; ** = P < .01; *** = P < .001. RSISP, routine screened infant surveillance program; CC, bronchoscopy for clinical concern; CF = Cystic Fibrosis.

BAL fluid PGP levels were below the lower limit of detection in 23 of the 24 (96%) non-CF control patients with the remaining patient having very low levels of the peptide (0.27 ng/ml; Fig. 1A). This patient was culture-positive for *P. aeruginosa* (though it is noteworthy that the 7 other non-CF control patients who were culture-positive for other organisms lacked PGP). Conversely, all but one of the CC-CF patients tested contained PGP in their BAL fluid above our threshold of detection with a median value (IQR) of 2.15 ng/ml (2.57 ng/ml) (Fig. 1A). Within this group, culture status had no significant impact on the levels of PGP, and no significant difference was detected in PGP levels in CC-CF children that were culture positive for *P. aeruginosa* versus those culture positive for other bacteria. PGP levels were below the lower limit of detection in the majority of RSISP-CF patients (89%) with the exception being the single culture-positive infant (*P. aeruginosa*) in whom substantial quantities of the peptide were detected (3.0 ng/ml). For the cohort overall, a positive correlation was observed between PGP concentration and neutrophil numbers (Fig. 1B). Whilst the concentration of IL-8 was significantly elevated in the BALF of the CC-CF group relative to the RSISP-CF and non-CF control patients (Fig. 1C), the separation was not as striking as observed with PGP, highlighting the potential of PGP as a potential severity biomarker for the former group. From the limited spirometric data available, PGP concentrations did not show a significant relationship with % predicted FEV_1_ in the year following bronchoscopy. Additionally, no significant correlation was observed between PGP concentration and slope of FEV_1_ decline during 5 years post bronchoscopy. No AcPGP was detectable in any of these pediatric samples.

### 3.4 Elevated PGP-generating enzymes in the BAL fluid of CC-CF children

**Fig. 2:**
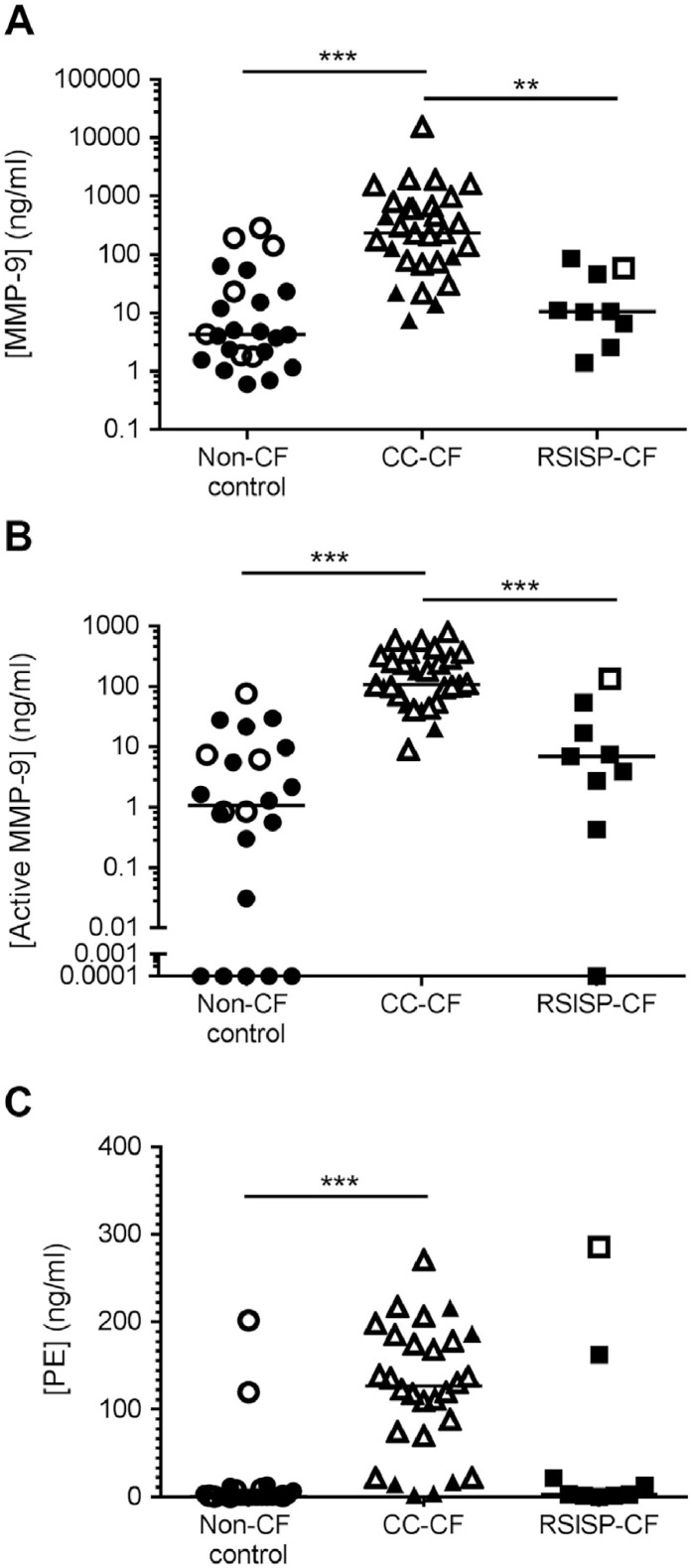
Elevated PGP-generating enzymes in the BAL fluid of older children with CF. Levels of total MMP-9 (A; n = 24 non-CF, 9 RSISP-CF, 29 CC-CF) and active MMP-9 (B; n = 22 non-CF, 9 RSISP-CF, 28 CC-CF) and PE (C; n = 24 non-CF, 9 RSISP-CF, 29 CC-CF) in the BAL fluid. Closed symbols represent patients that were culture negative and open symbols patients that were culture positive. The horizontal bar depicts the median of each group. Statistical significance between groups was tested using a Kruskal-Wallis test followed by a Dunns post-test. * = P < .05; ** = P < .01; *** = P < .001. RSISP, routine screened infant surveillance program; CC, bronchoscopy for clinical concern; CF = Cystic Fibrosis.

Levels of total (Fig. 2A) and active (Fig. 2B) MMP-9 were significantly elevated in CC-CF group compared to RSISP-CF patients and non-CF controls. In this CC-CF group, MMP-9 levels were also significantly higher in the culture positive than culture negative patients. In the entire cohort, there was a strong correlation between MMP-9 and airway neutrophilia (Supplementary Fig. 1A). Levels of PE showed a similar pattern to MMP-9, being significantly raised in CC-CF patients versus non-CF controls (Fig. 2C), demonstrating trends towards higher levels in the presence of infection and a relationship with neutrophilia (Supplementary Fig. 1B). It is pertinent that the one RSISP-CF patient that presented with BAL fluid PGP also had particularly pronounced PE levels (Fig. 2C). Whilst MMP-9 and PE levels correlated with PGP levels (Supplementary Fig. 1C and D), there were some non-CF control and RSISP-CF children that lacked PGP whilst possessing greater levels of MMP-9/PE than some CC-CF children. This suggests that elevated MMP-9/PE alone is insufficient to account for the striking augmentation in PGP levels observed in CC-CF children.

### 3.5 Reduced levels of extracellular LTA_4_H in the BAL fluid of CC-CF children

**Fig. 3:**
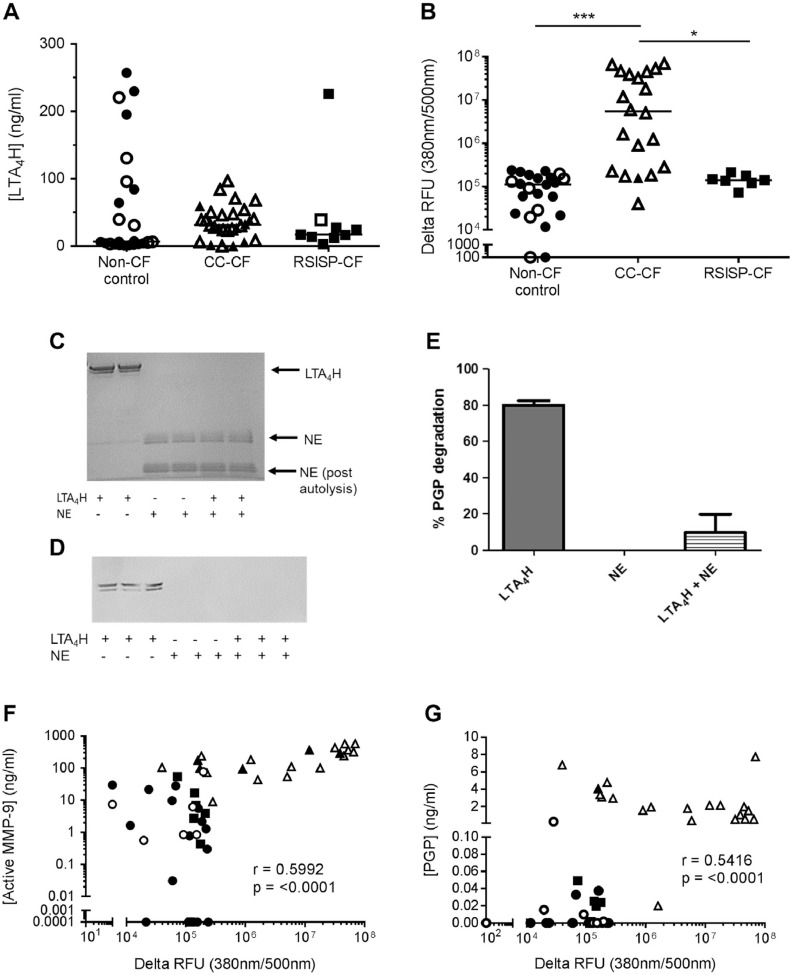
Neutrophil elastase can cleave LTA_4_H, abrogating its PGP-degrading activity. (A) Levels of LTA_4_H in the BAL fluid of non-CF controls (n = 24), RSISP-CF patients (n = 9) and CC-CF patients (n = 29), as determined by ELISA. (B) NE activity in the BAL fluid of non-CF controls (n = 23), RSISP-CF patients (n = 7) CC-CF patients (n = 20), as determined by fluorometric assay. (C-E) Recombinant LTA_4_H was co-incubated with recombinant neutrophil elastase (NE) for 2 h at 37 °C and cleavage of LTA_4_H assessed by Coomassie stained gel (C) and Western blot (D). (E) Recombinant LTA_4_H was co-incubated with recombinant NE for 2 h at 37 °C and capacity of LTA_4_H to degrade PGP subsequently determined by incubation with the peptide for 2 h and assessing PGP degradation by LC-MS/MS. (F) Correlation between levels of active MMP-9 and NE activity in the BAL fluid (n = 46: 22 non-CF, 7 RSISP-CF, 17 CC-CF). (G) Correlation between levels of PGP and NE activity in the BAL fluid (n = 50: 23 non-CF, 7 RSISP-CF, 20 CC-CF). Closed symbols represent patients that were culture negative and open symbols patients that were culture positive. For (A and B) the horizontal bar depicts the median of each group. Statistical significance between groups was tested using a Kruskal-Wallis test followed by a Dunns post-test and correlation analysis was performed using Spearman rank test. * = P < .05; ** = P < .01; *** = P < .001. RSISP, routine screened infant surveillance program; CC, bronchoscopy for clinical concern; CF = Cystic Fibrosis.

PGP persistence is a function of the relative activities of MMP-9/PE versus LTA_4_H. Extracellular LTA_4_H levels are substantially elevated during inflammatory episodes concomitant with MMP-9 and PE to ensure that PGP is efficiently degraded [4,9,10]. However, counter to expectations, CC-CF patients displayed comparable or even reduced levels of LTA_4_H in their BAL fluid relative to the non-CF controls (Fig. 3A). It is therefore feasible that the relative loss of LTA_4_H contributes to the PGP accumulation in older CF children. NE is a prominent protease implicated in the pathology of CF lung disease, and analysis of the LTA_4_H protein sequence revealed multiple potential NE cleavage sites. Accordingly, NE activity was significantly elevated in the CC-CF children (Fig. 3B). Incubation of recombinant LTA_4_H with recombinant active NE resulted in loss of LTA_4_H protein, as adjudged by Coomassie stained gel (Fig. 3C) and Western blot (Fig. 3D), and enzymatic PGP-degrading activity (Fig. 3E). It has recently been demonstrated that NE can activate MMP-9 [22] and could thus further perpetuate the PGP cycle of inflammation. Accordingly, levels of active MMP-9 correlated with NE activity (Fig. 3F). Thus NE activity, a surrogate marker for lung disease severity in CF children, can activate PGP-generating MMP-9 and destroy PGP-degrading LTA_4_H. Consequently, a significant correlation is observed between NE activity and PGP levels (Fig. 3G).

Whilst PGP degradation is mediated by the aminopeptidase activity of LTA_4_H in an extracellular environment, LTB_4_ is generated intracellularly by the epoxide hydrolase activity of LTA_4_H. Clearly, only extracellular LTA_4_H would be liable to neutrophil elastase degradation as seen in the CC-CF children, with the intracellular enzyme still capable of generating LTB_4_. Accordingly, we report elevated LTB_4_ levels in the BAL fluid of CC-CF children (Supplementary Fig. 2A).

### 3.6 An imbalance between PGP-generating and -degrading enzymes permits PGP accumulation in children with CF

**Fig. 4:**
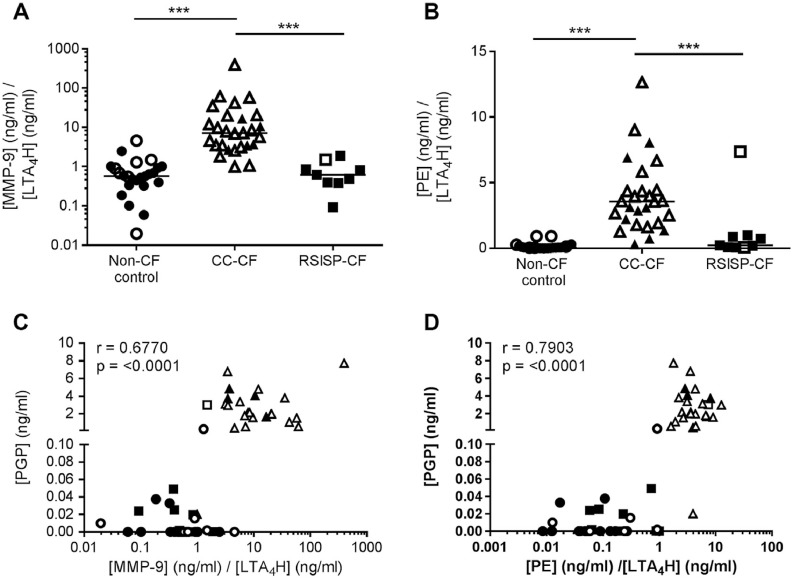
An imbalance between PGP-generating and -degrading enzymes permits PGP accumulation in CF children. Ratio of [MMP-9]/[LTA_4_H] (A) or [PE]/[LTA_4_H] (B) in the BAL fluid of non-CF controls (n = 24), RSISP-CF patients (n = 9) and CC-CF patients (n = 27), as determined by ELISA. (C) Correlation between the ratio of [MMP-9]/[LTA_4_H] versus [PGP] or (D) between the ratio of [PE]/[LTA_4_H] versus PGP (n = 56: 24 non-CF, 9 RSISP-CF, 23 CC-CF). Closed symbols represent patients that were culture negative and open symbols patients that were culture positive. For (A and B) the horizontal bar depicts the median of each group. Statistical significance between groups was tested using a Kruskal-Wallis test followed by a Dunns post-test and correlation analysis was performed using Spearman rank test. * = P < .05; ** = P < .01; *** = P < .001. RSISP, routine screened infant surveillance program; CC, bronchoscopy for clinical concern; CF = Cystic Fibrosis.

We rationalized that the relative amounts of PGP-generating and –degrading enzymes in each child ultimately defined the level of PGP able to persist. When the ratio of total or active MMP-9 to extracellular LTA_4_H levels/activity was determined, there was a clear and striking augmentation in the CC-CF children relative to non-CF controls and RSISP-CF patients (Fig. 4A; Supplementary Fig. 3A and B), rationalizing the clear separation in levels of PGP between the groups of children. When expressing the ratio of PE to LTA_4_H levels/activity there was once again the same clear distinction between groups (Fig. 4B; Supplementary Fig. 3C). It is noteworthy that the one RSISP-CF patient that was culture positive (*P. aeruginosa*) and possessed comparable levels of PGP relative to the older CC-CF children, also presented with a comparable PE: LTA_4_H ratio. Thus children that displayed a marked imbalance in the relative levels of PGP-generating to PGP-degrading enzymes were those that possessed PGP. Indeed, a strong positive correlation is observed when comparing the MMP-9: LTA_4_H ratio to PGP (Fig. 4C) or the PE: LTA_4_H ratio to PGP (Fig. 4D).

## 4 Discussion

Characteristic features of CF airways disease are chronic infection and the predominance of neutrophils in the airway lumen, which ultimately drive tissue damage and disease progression [[23], [24], [25], [26]]. Within weeks of birth some CF infants have been reported to present with infection and inflammation [25,[27], [28], [29]] and by adolescence inflammation and infection are uniformly present even in the absence of symptoms [30]. Debate continues as to whether the inflammation arises in response to infection or whether the CF airway is intrinsically pro-inflammatory. In this study, we questioned whether PGP could support neutrophilic inflammation in children with CF, including newborn infants without current or prior detectable infection.

We report that the vast majority of older children with CF (CC-CF) have substantial quantities of PGP in their BAL fluid, which correlated with the elevated neutrophil numbers in this population. Conversely, non-CF controls were largely devoid of BAL PGP, even though some were culture positive and presented with neutrophilia - supportive of the notion that PGP is normally efficiently degraded to limit neutrophilic inflammation and only accumulates in chronic diseases when this system fails [4,10]. PGP was below the limit of detection in the vast majority of newborn children with CF (RSISP-CF). Thus it would appear that the augmented PGP levels observed in the CC-CF group are not intrinsic to CF status but are instead acquired with disease progression. Intriguingly, the one RSISP-CF patient that was culture-positive displayed substantial quantities of the peptide. Despite small sample sizes, it is appealing to speculate that a combination of CF status and infectious insult is required to initiate PGP accumulation. In older children with CF, current culture status may be only partially relevant since they will have likely previously experienced multiple infective “triggers” leading to PGP accumulation and ensuing self-propagating cycle of neutrophilic inflammation.

A pertinent question is how PGP is able to accumulate in CC-CF children. We report that levels of PGP-generating enzymes MMP-9 and PE are elevated in the CC-CF group relative to non-CF controls and RSISP-CF patients. Levels of these enzymes were generally higher in children that were culture positive, potentially indicative of the greater neutrophilic infiltrate [4,10]. It is noteworthy, however, that release of PE from airway epithelia has also been reported in response to TLR4 stimulation via exosomes and that CF subjects colonized with *P. aeruginosa* demonstrate elevated exosome PE content [31] – potentially rationalizing the elevated levels of this enzyme with infection. Whilst MMP-9/PE levels were elevated in CC-CF patients, there are some non-CF control children that lacked PGP whilst possessing greater neutrophil numbers and levels of MMP-9/PE than some in the CC-CF group. This suggests that elevated MMP-9/PE alone was insufficient to drive PGP accumulation in CF children.

Extracellular LTA_4_H levels are normally augmented with neutrophilic inflammation concomitantly with MMP-9/PE to ensure PGP is efficiently degraded [4,9,10]. Whilst one would envisage that extracellular LTA_4_H levels should therefore be substantially higher in CC-CF children, this was not the case and levels were generally very low. NE is a protease that is most prominently implicated in the pathology of CF lung disease and a surrogate marker of disease severity in children with CF [[32], [33], [34], [35], [36]]. We demonstrate that NE can degrade LTA_4_H, abrogating its activity. Accordingly NE activity was augmented in CC-CF children, supportive of the notion that it may contribute to the loss of LTA_4_H in these patients. We have previously demonstrated that PGP not only drives neutrophil recruitment but also promotes neutrophil NE release [6,10]. In light of current findings, this would result in degradation of extracellular LTA_4_H, prevention of PGP degradation and would re-enforce the whole vicious cycle of protease/matrikine driven inflammation. Furthermore, it has recently been demonstrated that NE can activate MMP-9 [22] thus further perpetuating this PGP cycle.

It would seem that PGP is able to accumulate in older children with CF as a consequence of elevated PGP-generating enzymes and a synchronized reduction in PGP-degrading LTA_4_H to in essence create the perfect storm. Accordingly, MMP-9: LTA_4_H or PE: LTA_4_H ratios were substantially greater in this CC-CF group than any other children and correlated strongly with PGP levels. The one exception being the one RSISP-CF patient that was culture positive and presented with substantial quantities of PGP – this infant showed a spike in PGP- displayed MMP-9: LTA_4_H and PE: LTA_4_H ratios more in keeping with the older CF children that also possessed PGP. Thus we would speculate that CFTR dysfunction is not directly driving the PGP accumulation observed in the CC-CF children, but rather that the persistent inflammation and infectious insults in the CF airways creates an environment that subverts the PGP pathway. Specifically, elevated NE levels in the CF airways destroys extracellular LTA_4_H, and thus when infections spike neutrophilic inflammation and release of PGP-generating enzymes, the liberated PGP cannot be efficiently degraded. Accumulated PGP would subsequently function to exacerbate inflammation by perpetuating a vicious circle of neutrophilic inflammation. We have previously demonstrated that a failure of LTA_4_H to degrade PGP in response to a normally self-limiting pulmonary bacterial infection, resulted in a greater neutrophilic exacerbation [4,10]. We speculate that this is essentially what is occurring in the CF lung whereby the LTA_4_H system is defective and thus upon infectious challenge, PGP generation spikes but the peptide is unable to be efficiently degraded. Recently, we have demonstrated in a mouse model of allergic airways disease that a failure to degrade PGP directly, and independently of its action on neutrophils, elicited pathological epithelial remodelling and mucus hypersecretion [9]. Given that structural airway wall changes are a hallmark feature of CF that begin in early life [37], then it is tempting to speculate that PGP could potentially be an instigator of this remodelling.

Whilst PGP degradation is mediated by the aminopeptidase activity of LTA_4_H in an extracellular environment, LTB_4_ is generated intracellularly by the epoxide hydrolase activity of LTA_4_H. Clearly, only extracellular LTA_4_H would be liable to neutrophil elastase degradation as seen in the CC-CF children, with the intracellular enzyme still capable of generating LTB_4_. Accordingly, we report elevated LTB_4_ levels in the BAL fluid of CC-CF children. Thus within this CC-CF group, the pro-inflammatory activity of LTA_4_H is functional but its anti-inflammatory activity is not, enabling accumulation of both LTB_4_ and PGP. Our findings are in agreement with previous studies describing increased LTB_4_ levels in CF airways [38]. Furthermore, the levels of pro-resolving eicosanoid lipoxin A_4_ (LXA_4_) are reduced in CF lung disease owing to reduced expression of the enzyme 15-lipoxygenase, giving rise to a LTB_4_ /LXA_4_ ratio that favours inflammation and impedes resolution [39,40]. Celtaxsys have looked to ameliorate LTB_4_-driven inflammation in CF through administration of their LTA_4_H inhibitor, Acebilustat. Encouragingly, this resulted in a modest reduction in CF exacerbations in a recent phase IIb trial. It could be argued that Acebilustat would inadvertently lead to PGP accumulation, which in turn could mask some of the benefits of ameliorating LTB_4_-driven inflammation [41]. However, our current study demonstrates that PGP degradation by LTA_4_H is already aberrant in CC-CF children, potentially owing to degradation of LTA_4_H by neutrophil elastase, and thus it is feasible that any potential adverse effects of Acebilustat in inhibiting PGP degradation by LTA_4_H would be negligible in CF.

Whilst IL-8 concentrations were also significantly elevated in older children with CF relative to newborn children with CF and non-CF controls, the separation in IL-8 levels between these patient groups was not as clearly defined or absolute as observed with PGP. This highlights the potential of PGP as a biomarker, superior to other neutrophil surrogate markers, for CF lung disease progression. Clearly, there are challenges with utilization of LC-MS/MS for biomarker assessment, including the high initial cost of the equipment, the high complexity of the instrumentation's operation and maintenance and the relatively low sample throughput. Nonetheless, LC-MS/MS has now become a widespread technology and routine analysis within clinical laboratories, and it is realistic to consider use of PGP as a biomarker for evaluation of CF disease progression.

There are some inevitable limitations associated with this study. The number of newborn children within the RSISP-CF group is relatively small, whilst the CC-CF group encompassed a relatively diverse range of clinical situations combining any patients older than 4 months who were having a bronchoscopy because of clinical deterioration. An associated potential caveat to these findings is that patients within the older CC-CF group were unstable and bronchoscopy was performed due to clinical concern, thus potentially limiting comparison to stable CF patients. This is an unavoidable limitation of our study given that routine surveillance bronchoscopy beyond 4 months of age has not been part of CF care at our centre. The variability of PGP with change in clinical stability in older children with CF is therefore an important question that requires further exploration. It is also feasible that medications differentially prescribed to patients could impact on the PGP pathway. Previous studies in the context of COPD have suggested that treatment of patients with macrolides [42] or Roflumilast [43] can reduce sputum PGP levels, particularly with increased duration of therapy. A proportion of the CC-CF children included in our study had received azithromycin, however this had no significant impact on PGP levels in the BAL fluid. Finally, our analysis has focused upon host-derived enzymes central to the PGP pathway, but it is feasible that bacteria express related enzymes with primitive PGP-generating or –degrading activities. Whilst beyond the scope of this study, it would be interesting in future to ascertain whether any bacterial species prevalent within the CF lung produce enzymes with the capacity to generate or degrade PGP and consequently the physiological relevance of these putative enzymes in defining protection and pathology.

PGP and its acetylated variant, AcPGP, have been reported in adult CF patients at baseline and during exacerbations, with levels of the peptides depreciating with inpatient therapy [3]. We didn't observe a significant relationship between PGP concentration and % predicted FEV_1_ in the year following bronchoscopy, or between PGP and rate of decline in FEV_1_ in the 5 years following bronchoscopy, in the patients for whom this measurement was available. The group is likely underpowered for analyses of this highly variable outcome measure, and also included children of variable ages, who may not be directly comparable. Furthermore, assessment of FEV_1_ was not close enough to the bronchoscopy to reflect acute changes in lung function. A final limitation is that at our centre, an annual assessment is not cancelled if the patient is clinically unwell, so values may not reflect their optimal clinical status. Unfortunately, the use of Lung Clearance Index was not part of routine clinical assessment at the time when the majority of these patient samples were collected, and so this data isn't available. It was surprising, given the abundance of AcPGP previously reported in adult CF patients [3], that none was present in CF children. Reactive aldehydes, such as acrolein, can acetylate PGP to generate AcPGP [5]. Acrolein can be generated during severe inflammation [44,45] and one could rationalize that with increasing age and recurrent and persistent inflammation in the CF lung, acrolein could accumulate and exacerbate inflammation via conversion of PGP to AcPGP.

## 5 Conclusions

In conclusion, this study has demonstrated that PGP accumulates in the CF lung during early life, as a consequence of a striking imbalance between PGP-generating (MMP-9 and PE) and –degrading (LTA_4_H) enzymes; with the latter being targeted for proteolytic degradation in the CF lung. We speculate that infectious insult is a key event by augmenting PGP generation in a context where it cannot be efficiently degraded, and in so doing elicit a self-sustaining vicious cycle of inflammation. The accumulated PGP can subsequently promote persistent airway neutrophilia and protease imbalance that are characteristic features of CF.

## Author contributions

RJS designed, executed (along with CJP, DFP and MMF) and interpreted the experiments and prepared the manuscript; JCD, AB and EWFWA led the clinical fellows (TH, NR, HT, SB, RT) acquiring the samples and clinical data; the fellows also performed cytospin analysis of BAL. JCD assisted with interpretation of the experiments and preparation of the manuscript. AT acquired and interpreted data within the manuscript and contributed discussions throughout the work. PLJ aided with mass spectrometry, and PLJ, AG, JEB and CML contributed discussions throughout the work.

## Conflict of interests

ART, PLJ, TNH, NR, HLT, SB, RT, EWFWA, AG, JEB, AB and RJS have no competing interests. JCD has financial relationships with Vertex, Proteostasis, PTC, Pulmocide, Bayer, Boehringer Ingelheim, Novartis and Enterprise Therapeutics, all independent from submitted work. CML has received funding from Janssen Biotech Inc. independent from the submitted work.

## Appendix A Supplementary data

Supplementary material Image 1
