# Supplementary material for: Abnormal pro-gly-pro pathway and airway neutrophilia in pediatric cystic fibrosis
Source: J Cyst Fibros. 2020 Jan;19(1):40–8. doi: 10.1016/j.jcf.2019.05.017 (PMC7001103; doi:10.1016/j.jcf.2019.05.017)
Supplement: Supplementary file 1 — Supplementary material [file mmc1.docx]

**Abnormal Pro-Gly-Pro pathway and airway neutrophilia in pediatric cystic fibrosis.**

Andrew R. Turnbull, Chloe J. Pyle, Dhiren F. Patel, Patricia L. Jackson, Tom N. Hilliard, Nicolas Regamey, Hui-Leng Tan, Sarah Brown, Rebecca Thursfield, Christopher Short, Megan Mc Fie, Eric W.F.W. Alton, Amit Gaggar, J. Edwin Blalock, Clare M. Lloyd, Andrew Bush, Jane C. Davies, Robert J. Snelgrove.

**SUPPLEMTARY MATERIAL**

**Supplementary Materials and Methods**

*Flexible bronchoscopy:* BAL was performed using three aliquots of 1 ml/kg 0.9% saline at room temperature instilled separately into one or two lobes (usually the right middle lobe and the clinically most affected lobe), and the returns pooled.

*BAL fluid analysis:* Aliquots of supernatant were frozen at -80^O^C until used. Cells were counted and differentials assessed on cytospin preparations using May-Gruenwald-Giemsa staining. Bacterial and fungal growth was assessed with standard CF-specific culture techniques by the hospital Clinical Microbiology Laboratory. Data were not available for viral detection.

*PGP degradation experiments:* Bronchoalveolar lavage fluid (diluted 1/10 in PBS) was incubated with 0.4mM PGP at 37^o^C in 5% CO_2_ for varying periods of time. Concentrations of PGP remaining were subsequently quantified by ESI-LC/MS/MS (as discussed below) by comparison with PGP standards. The amount of peptide degraded was determined relative to control samples of 0.4mM PGP alone. PGP degradation was also assessed by measurement of free proline released and its ensuing reaction with Ninhydrin (as discussed below).

*ESI-LC/MS/MS for PGP detection:* For peptide quantification in BAL fluid, PGP and AcPGP were measured using a MDS Sciex API-4000 spectrometer (Applied Biosystems, Foster City, CA) equipped with a Shimadzu HPLC (Columbia, MD). For peptide quantification from degradation experiments, PGP and AcPGP were measured using a Thermo Accela Pump and Autosampler coupled to a Thermo TSQ Quantum Access. HPLC was done using a 2.0 x 150 mm Jupiter 4u Proteo column (Phenomenex, Torrance, CA) with A: 0.1% HCOOH and B: MeCN + 0.1% HCOOH: 0 min-0.5 min 5% buffer B/95% buffer A, then increased over 0.5-2.5 min to 100% buffer B/0% buffer A. Background was removed by flushing with 100% isopropanol / 0.1% formic acid. Positive electrospray mass transitions were at 270-70, 270-116 and 270-173 for PGP and 312-140 and 312-112 of AcPGP. Peak area was measured, and PGP/AcPGP peptide concentrations were calculated using a relative standard curve method.

*Measurement of free proline:* Aliquots from PGP degradation experiments were diluted 1 in 10 in PBS (to a final volume of 250µl). Glacial acetic acid (250µl) was then added, followed by 250μl of ninhydrin solution (25 mg/ml in acetic acid/6 M phosphoric acid; heated at 70°C to dissolve). The reaction mixture was heated at 100°C for 60 minutes, allowed to cool to room temperature and the proline containing fraction extracted with 500µl of toluene and optical density measured at 520 nm.

*LTA_4_H degradation by NE:* Recombinant human LTA_4_H (final concentration 20µg/ml; Cayman Chemical, Ann Arbor, USA) and NE (final concentration 20µg/ml; Abcam, Cambridge, UK) were incubated alone or in combination in a final volume of 100 µl PBS at 37°C in 5% CO_2_ for 2 hrs. To assess LTA_4_H protein levels via Western blot, samples were electrophoresed through reducing SDS-polyacrylamide gels (Life Technologies Ltd, Paisley, UK) and electroblotted onto nitrocellulose membranes (Life Technologies Ltd, Paisley, UK). Membranes were blocked in TBST containing 5% milk for 2 hrs at 22°C and incubated with goat polyclonal anti-LTA4H (0.2 µg/ml, Santa Cruz Biotechnology, Santa Cruz, CA) diluted in 5% milk/TBST overnight at 4°C. Once washed, membranes were incubated with donkey anti-goat IgG-HRP (1/5000, Santa Cruz Biotechnology, Santa Cruz, CA) and reactivity was detected using ECL chemiluminescent kits (Pierce, Rockford, IL) according to manufacturer’s directions and chemiluminescence measured using a Thermo Scientific MYECL Imager (Thermoscientific, Rockford, IL). To assess LTA_4_H PGP-degrading activity, samples were diluted 1/20 and admixed with 0.4mM PGP at 37^o^C in 5% CO_2_ for 2 hrs. Loss of PGP was subsequently assessed by ESI-LC/MS/MS and generation of free proline by reaction with Ninhydrin, as described above.

For clear visualization of LTA_4_H and NE on Coomassie stained gels it was necessary to increase the relative concentrations of the enzymes. Accordingly, recombinant human LTA_4_H (final concentration 100µg/ml) and NE (final concentration 100µg/ml) were incubated alone or in combination in a final volume of 100 µl PBS at 37°C in 5% CO_2_ for 2 hrs. Samples were subsequently electrophoresed through reducing SDS-polyacrylamide gels, and stained with SimplyBlue Safe Stain (Life Technologies Ltd, Paisley, UK).

**Supplementary Figures.**

**
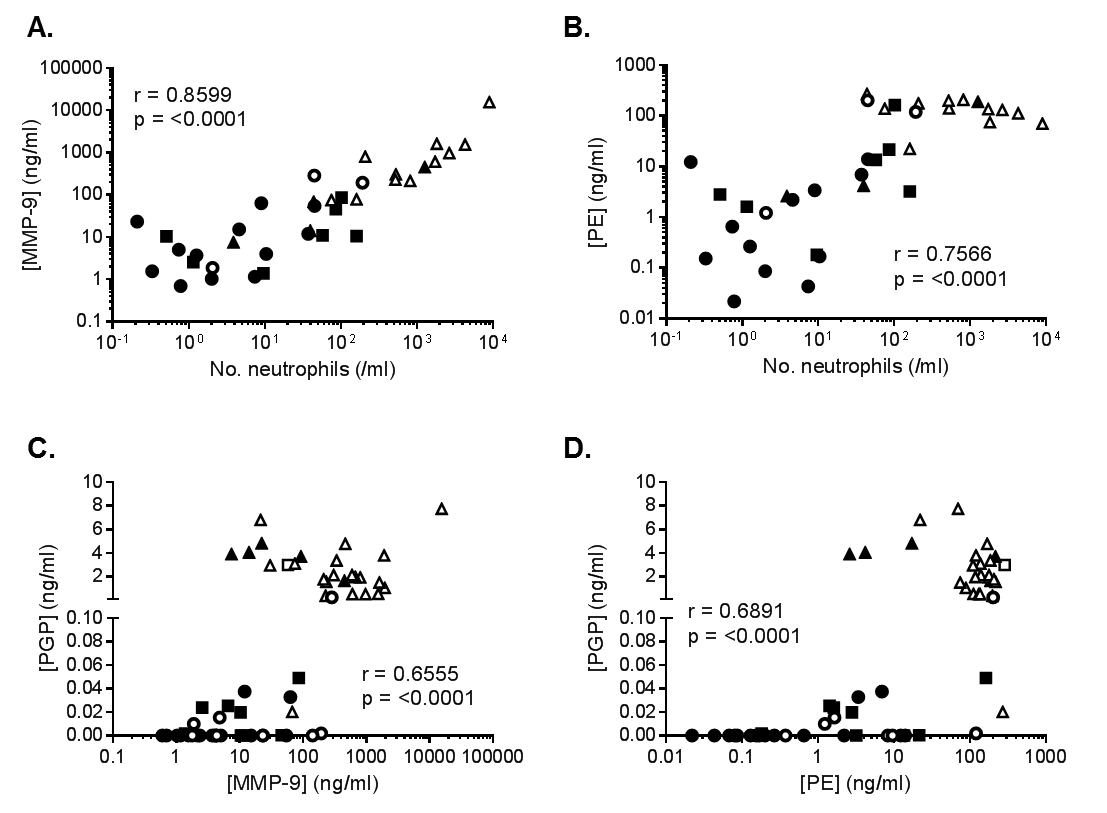
**

**Supplementary Figure 1. MMP-9 and PE correlate with neutrophil numbers and PGP levels in BAL fluid.**

Correlation between levels of MMP-9 (A) or PE (B) and neutrophil numbers in the BAL fluid (n=38: 17 non-CF, 7 RSISP-CF, 15 CC-CF). Correlation between levels of MMP-9 (C) or PE (D) and PGP in the BAL fluid (n=58: 24 non-CF, 9 RSISP-CF, 25 CC-CF). Circles depict non-CF control group, triangles depict CC-CF group and squares depict RSISP-CF group. Closed symbols represent patients that were culture negative and open symbols patients that were culture positive. Correlation analysis was performed using Spearman rank test. RSISP, routine screened infant surveillance program; CC, bronchoscopy for clinical concern; CF= Cystic Fibrosis.

**
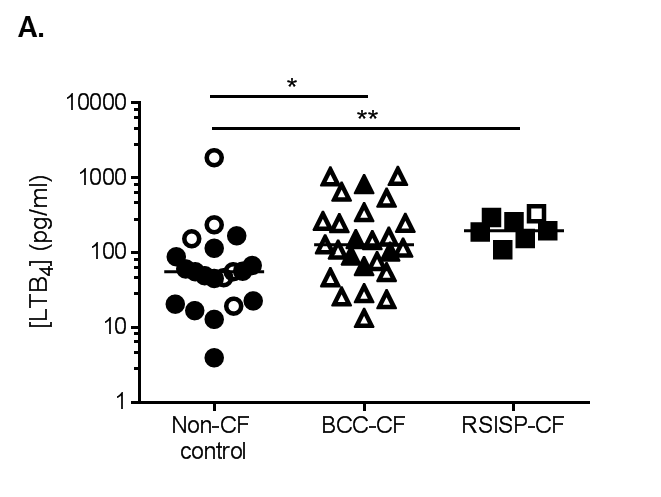
**

**Supplementary Figure 2. Elevated LTB_4_ in the BAL fluid of older children with CF.**

(A) Levels of LTB_4_ in the BAL fluid of non-CF controls (n=24), RSISP-CF children (n=9) and CC-CF children (n=25), as determined by immunoassay. Closed symbols represent patients that were culture negative and open symbols patients that were culture positive. The horizontal bar depicts the median of each group. Statistical significance between groups was tested using a Kruskal-Wallis test followed by a Dunns post-test. * = P<0.05; ** = P<0.01; *** = P<0.001. RSISP, routine screened infant surveillance program; CC, bronchoscopy for clinical concern; CF= Cystic Fibrosis.

**
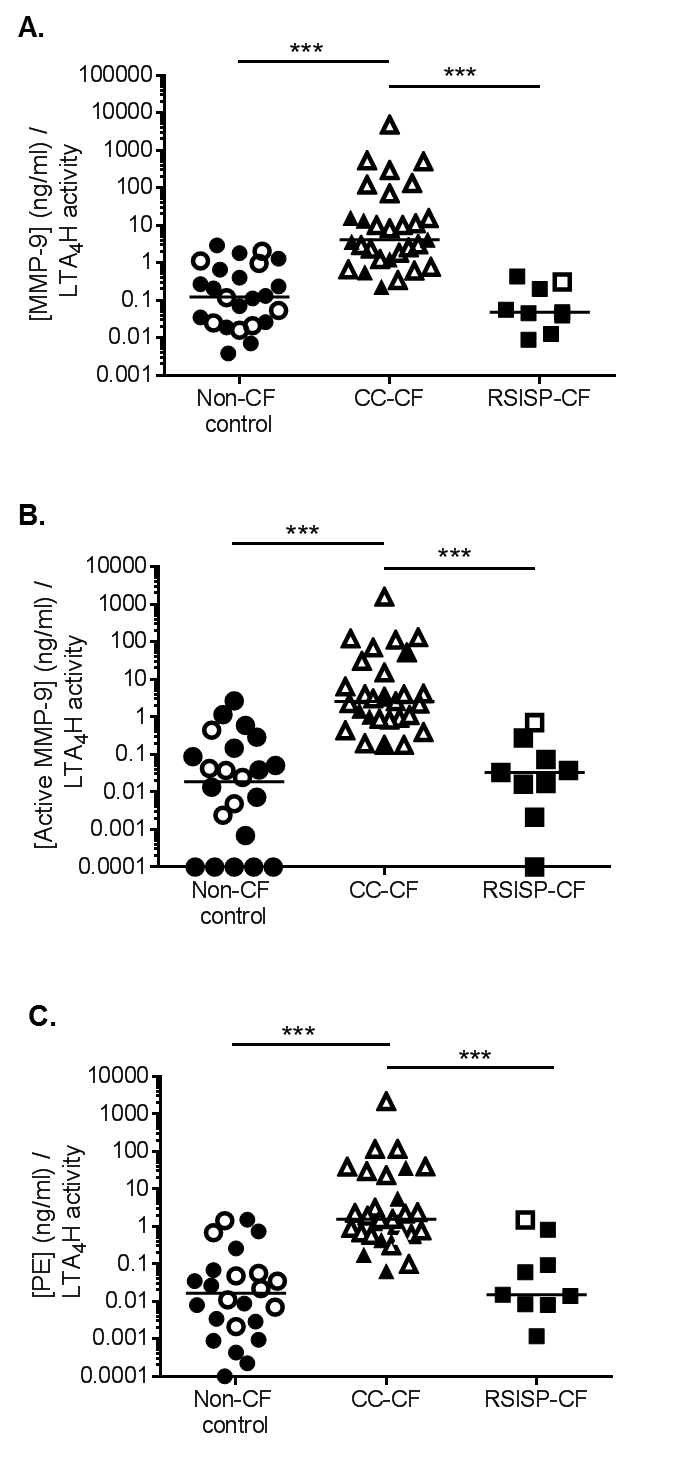
**

**Supplementary Figure 3. An imbalance between PGP-generating enzymes and PGP-degrading activity permits PGP accumulation in CF children.**

Ratio of total MMP-9 protein /LTA_4_H activity (A) or active MMP-9 /LTA_4_H activity (B) or PE protein /LTA_4_H activity (C) in BAL fluid of 24 non-CF, 9 RSISP-CF and 29 CC-CF patients. Closed symbols represent patients that were culture negative and open symbols patients that were culture positive. The horizontal bar depicts the median of each group. Statistical significance between groups was tested using a Kruskal-Wallis test followed by a Dunns post-test. * = P<0.05; ** = P<0.01; *** = P<0.001. RSISP, routine screened infant surveillance program; CC, bronchoscopy for clinical concern; CF= Cystic Fibrosis.
